# Machine Learning for the Prediction of Procedural Case Durations Developed Using a Large Multicenter Database: Algorithm Development and Validation Study

**DOI:** 10.2196/44909

**Published:** 2023-09-08

**Authors:** Samir Kendale, Andrew Bishara, Michael Burns, Stuart Solomon, Matthew Corriere, Michael Mathis

**Affiliations:** 1 Department of Anesthesia, Critical Care & Pain Medicine Beth Israel Deaconess Medical Center Boston, MA United States; 2 Department of Anesthesia and Perioperative Care University of California, San Francisco San Francisco, CA United States; 3 Bakar Computational Health Sciences Institute University of California San Francisco San Francisco, CA United States; 4 Department of Anesthesiology University of Michigan Medical School Ann Arbor, MI United States; 5 Department of Anesthesiology The University of Texas Health Science Center at San Antonio San Antonio, TX United States; 6 Department of Surgery Section of Vascular Surgery University of Michigan Medical School Ann Arbor, MI United States; 7 Center for Computational Medicine and Bioinformatics University of Michigan Medical School Ann Arbor, MI United States

**Keywords:** medical informatics, artificial intelligence, AI, machine learning, operating room, OR management, perioperative, algorithm development, validation, patient communication, surgical procedure, prediction model

## Abstract

**Background:**

Accurate projections of procedural case durations are complex but critical to the planning of perioperative staffing, operating room resources, and patient communication. Nonlinear prediction models using machine learning methods may provide opportunities for hospitals to improve upon current estimates of procedure duration.

**Objective:**

The aim of this study was to determine whether a machine learning algorithm scalable across multiple centers could make estimations of case duration within a tolerance limit because there are substantial resources required for operating room functioning that relate to case duration.

**Methods:**

Deep learning, gradient boosting, and ensemble machine learning models were generated using perioperative data available at 3 distinct time points: the time of scheduling, the time of patient arrival to the operating or procedure room (primary model), and the time of surgical incision or procedure start. The primary outcome was procedure duration, defined by the time between the arrival and the departure of the patient from the procedure room. Model performance was assessed by mean absolute error (MAE), the proportion of predictions falling within 20% of the actual duration, and other standard metrics. Performance was compared with a baseline method of historical means within a linear regression model. Model features driving predictions were assessed using Shapley additive explanations values and permutation feature importance.

**Results:**

A total of 1,177,893 procedures from 13 academic and private hospitals between 2016 and 2019 were used. Across all procedures, the median procedure duration was 94 (IQR 50-167) minutes. In estimating the procedure duration, the gradient boosting machine was the best-performing model, demonstrating an MAE of 34 (SD 47) minutes, with 46% of the predictions falling within 20% of the actual duration in the test data set. This represented a statistically and clinically significant improvement in predictions compared with a baseline linear regression model (MAE 43 min; *P*<.001; 39% of the predictions falling within 20% of the actual duration). The most important features in model training were historical procedure duration by surgeon, the word “free” within the procedure text, and the time of day.

**Conclusions:**

Nonlinear models using machine learning techniques may be used to generate high-performing, automatable, explainable, and scalable prediction models for procedure duration.

## Introduction

### Background

Across health care settings, anesthesiologist staffing and resources are commonly allocated based on procedure volume, concurrency, complexity, and projected duration [1,2]. Although preparing allocations is often done far in advance, depending on institutional processes, daily scheduling requires accurate information regarding recovery room availability as well as surgical, anesthesiology, and nurse staffing, all of which directly rely on accurate determination of procedure duration. More accurate prediction of procedure duration may allow for more effective assignment of procedure rooms, more efficient scheduling of cases (eg, staggering procedure rooms for surgeons with multiple cases), more predictable hours for involved staff, and clearer patient communication. Firmer understanding also relates to the high cost of running procedure rooms and maintaining optimal procedure room use. In addition, inaccurate estimates of case length affect patient care because they lead to gaps within block schedules that are not optimally used. This can lead to add-on cases not being completed in a timely manner as well as bed control issues in the inpatient setting or discharge issues in the outpatient setting. To manage procedure time, most institutions use either surgeon-directed procedure durations or procedure durations based on historical averages [3,4], which can be frequently inaccurate [1,2]. Because of the complexity of the problem and the inclusion of large numbers of features with potential interactions, linear regression methods to predict procedure durations have demonstrated varying levels of success [5-9]. Machine learning approaches have been proposed to mitigate this issue. In short, machine learning aims to extract patterns of knowledge from data, the benefit being the ability to process large volumes of disparate data, exploring potentially nonlinear interactions that may challenge the required assumptions of conventional analysis. Nonetheless, current studies have been limited to single or few institutions, smaller sample sizes (between 400 and 80,000 cases), specific surgical subpopulations (robotic [10], colorectal [11], and pediatric [12]), or the use of proprietary algorithms [10-16]; for example, the study by Lam et al [11] was multicenter but had approximately 10,000 colorectal cases. The studies by Tuwatananurak et al [13] and Rozario and Rozario [14] used proprietary tools, which may be useful for adoption but do not permit the same level of transparency or explainability as other methods. The included features varied significantly across previous studies.

### Objectives

Given the limitations of previous studies and the dependency of machine learning performance on training set size and heterogeneity, we developed a machine learning algorithm derived from a large multicenter data set for a more accurate prediction of surgical procedure duration compared with historical averages of procedure time. We hypothesized that a machine learning algorithm derived from a large multicenter data set with >1 million procedures would more accurately predict surgical procedure duration than a baseline linear regression approach. Using an explainable machine learning–based algorithm, the results can provide additional valuable insight regarding procedure duration and variability. The clinical objective of this protocol was to determine whether a machine learning algorithm scalable across multiple centers could make estimations of case duration within a tolerance limit because there are substantial resources required for procedure room functioning that relate to case duration.

## Methods

### Ethics Approval

We obtained institutional review board approval for this multicenter observational study from New York University (NYU) Langone Health, New York, NY (S19-01451), and the requirement for written informed consent was waived by the institutional review board.

### Study Design

We followed multidisciplinary guidelines for reporting machine learning–based prediction models in biomedical research [17,18]. Study outcomes, data collection, and statistical methods were established a priori and presented and approved at a multicenter peer review forum on January 13, 2020, before data analyses [19].

### Data Source

Data were provided by the Multicenter Perioperative Outcomes Group (MPOG). Within this research consortium, data from enterprise and departmental electronic health record systems are routinely uploaded to a secure centralized database. Methods for local electronic health record data acquisition, validation, mapping to interoperable universal MPOG concepts, and secure transfer to the coordinating center have been previously described [20] and used in multiple published studies [21-24]. In brief, each center uses a standardized set of data diagnostics to evaluate and address data quality on a monthly basis. Random subsets of cases are manually audited by a clinician at each center to assess, and attest to, the accuracy of data extraction and source data. At each institution participating in the MPOG, at the time of clinical onboarding (ie, when a new site joins the MPOG), a site-level data audit that involves hundreds of cases is initially performed until reaching a level of accuracy acceptable to the local site data quality reviewer. After this iterative process, the onboarded sites undergo a manual review of a minimum of 5 cases per month to ensure that changes in clinical and documentation practice patterns do not meaningfully degrade data quality over time [20]. All institutions were in the United States and ranged from community hospitals to large academic centers. A list of included centers is provided in Table S1 of Multimedia Appendix 1.

### Study Population

The study population included adult and pediatric patients who underwent procedures requiring anesthesiologist care between June 1, 2016, and November 30, 2019. Labor epidurals, labor analgesia, and procedures lacking relevant time points (patient-in-room duration) or provider information (surgeon and anesthesia staff identities anonymized) were excluded. Other missing data were handled as described in the following subsections.

### Primary Outcome

The primary outcome was procedure duration. Procedure duration was defined according to the precomputed *procedure room duration* electronic health record phenotype, interoperable across a wide variety of electronic health record vendors. The implications of over- and underpredicting the length of the procedure cannot be universally defined because this will be dictated by institutional policy and culture, but, broadly, overprediction (predicting a longer case than actual duration) may result in underuse of a given surgical block time, whereas underprediction (predicting a shorter case than actual duration) may result in inadequate staffing models.

### Basic Model Features

The features considered were determined by availability within the MPOG data and included certain patient characteristics such as sex, height, weight, and BMI; medical comorbidities; allergies; baseline vital signs; functional status; home medications; the day of the week; procedure text; procedure room type; anesthesia techniques; case times and durations; and deidentified institution and staff identities. Features were selected for modeling based on a review of the existing literature as well as by clinical and managerial experience [6,8,25]. Table 1 indicates the features that were ultimately selected to be used for the primary model and sensitivity analysis models using data available at varying time points relative to the start of the procedure. The primary model used features only available at the time the patient arrived in the procedure room. Of the 2 secondary models, one used features restricted to those available at the time of surgical scheduling, and the other used features expanded to those available after patient arrival to the procedure room up to the time of procedure start. This is described further in the Sensitivity Analyses subsection.

**Table 1:** Summary of prediction model features.

Model feature	Included in *Time of Scheduling* model (secondary model)	Included in *Time of Patient in OR*^a^ model (primary model)	Included in *Time of Surgical Incision* model (secondary model)
Case duration	✓	✓	✓
Holiday	✓	✓	✓
Weekend	✓	✓	✓
Surgical service	✓	✓	✓
Surgical procedure text	✓	✓	✓
Anonymized surgeon identity	✓	✓	✓
Patient age	✓	✓	✓
Patient BMI	✓	✓	✓
Location type (acute care hospital, mixed use OR, freestanding ambulatory surgical center, etc)	✓	✓	✓
Institution	✓	✓	✓
Preoperative comorbidities, including arrhythmia, CHF^b^, CAD^c^, HTN^d^, MI^e^, COPD^f^, diabetes, renal failure, liver disease, coagulopathy, cancer, and psychiatric illness (based on MPOG^g^ phenotype or preoperative anesthesia H&P^h^)	✓	✓	✓
Number of allergies	✓	✓	✓
Preoperative laboratory values, including creatinine, hemoglobin, albumin, INR^i^, and glucose levels		✓	✓
Preoperative baseline blood pressure		✓	✓
Preoperative existing airway		✓	✓
Anonymized anesthesia staff		✓	✓
ASA^j^ physical status score		✓	✓
Type of anesthesia			✓
Type of airway management			✓
Presence of nerve block			✓
Presence of neuraxial block			✓
Number of intravenous lines at the time of surgical procedure start			✓
Presence of arterial line at the time of surgical procedure start			✓
Time from patient arrival in the OR to anesthesia induction end			✓
Time from anesthesia induction end to surgical incision			✓

^a^OR: operating room.

^b^CHF: congestive heart failure.

^c^CAD: coronary artery disease.

^d^HTN: hypertension.

^e^MI: myocardial infarction.

^f^COPD: chronic obstructive pulmonary disease.

^g^MPOG: Multicenter Perioperative Outcomes Group.

^h^H&P: history and physical examination.

^i^INR: international normalized ratio.

^j^ASA: American Society of Anesthesiologists.

### Experience and Historical Features

Additional features were derived from surgical staff and institution identity (Textbox 1).

Derived experience and historical features were computed on a monthly basis from the earliest available date to the month before a given procedure; for example, procedures for the month of February 2019 would include derived features from June 2016 (the first available date in the data set) until January 2019. Procedure-wise features (eg, features derived from the first available date until the actual date of the procedure) were not included in the data set owing to computational processing power cost. Only the primary surgeon identity, anesthesiologist identity, and current procedural terminology (CPT) code were used in feature engineering. Density features were included to account for surgeons or institutions that were not included from the earliest date in the MPOG data set; for example, a surgeon who performed 20 procedures in 2 months would have the same density as a surgeon who performed 40 procedures in 4 months to mitigate model bias attributable to surgeons or institutions first appearing in the data set beyond the start date of the data set. Surgeon and institution experience are limited by the start date of the data set and would not account for experience before this start date. The same surgeon would have 2 different identities at different facilities because surgeons may fundamentally do different things based on the hospital they are practicing at, given the resources available to them at that specific hospital and practice patterns that are generally followed at that hospital.

**Additional features.**

- Surgeon experience: total number of procedures performed by surgeon
- Surgeon procedure experience: total number of a given procedure (by anesthesiology current procedural terminology [CPT] code) performed by surgeon
- Institutional procedure experience: total number of a given procedure performed at an institution
- Historical procedure duration: historical mean duration of a given procedure (by CPT code)
- Historical procedure duration by institution: historical mean duration of a given procedure at an institution
- Historical procedure duration by surgeon: historical mean duration of a given procedure by surgeon
- Surgeon total density: surgeon experience divided by time since surgeon’s first procedure
- Surgeon procedure density: surgeon procedure experience divided by time since surgeon’s first procedure
- Institutional procedure density: institutional procedure experience divided by time since institution’s first procedure

### Procedure Text Features

Although it was an option to include only the machine learning–generated anesthesia CPT code as a feature, it was felt that these codes lack the granularity that would be needed for more accurate prediction in this context. *Procedure text* refers to the name of the surgical procedure as booked by the surgeon. As the data set was being generated from a variety of institutions, *procedure text* may refer to either a scheduled procedure or a performed procedure and may vary in descriptiveness based on surgeon preference and institutional culture. Examples of *procedure text* may be “laparoscopic cholecystectomy with intraoperative cholangiogram” or “posterior cervical fusion C3-C7.” Natural language processing was used to convert text into a form usable by machine learning. Through a manual review of the corpus, common misspellings were corrected, and the 5 most common abbreviations were expanded (as detailed in Multimedia Appendix 1 [refer to R Code for Data Processing]). To decrease vocabulary size, text was standardized through the removal of punctuations and common stop words (eg, “a,” “an,” and “the”). Additional words deemed likely to be nondeterminative of procedure duration, such as “right” and “left,” were also preemptively removed. After text processing, term matrices were created with 1- and 2-word n-grams. Term frequency–inverse document frequency was used to transform text into numerical values. Because of the vastness of the corpus, but also to retain as many relevant terms as possible, terms with document frequency >0.995 were removed because these terms likely did not contain important information. Similar processing of procedure text for machine learning has been described in other published works [21]. The code for natural language processing is provided along with other data processing code in Multimedia Appendix 1 (refer to R Code for Data Processing).

### Power Analysis

Previous studies estimating procedure duration have used between 400 and 80,000 cases [9,10,12,13,26]. On the basis of experience and other comparable machine learning problems, we estimated that at least 100,000 cases encompassing a wide range of surgical procedure types would be adequate. On the basis of initial cohort size queries, there were >100,000 cases available for training, testing, and validation. A greater number of cases with a wider diversity in procedure types leads to a stronger machine learning model with less overfitting and ultimately greater generalizability [27,28].

### Data Preprocessing

All data were examined for missingness and veracity; cases with missing procedure duration and surgeon or anesthesia staff identities were eliminated. Outlier cases with durations of >1440 minutes were removed. Any feature missing >40% of the values or missing from >40% of the institutions was excluded. The remaining features were considered qualifying data. Different machine learning algorithms automatically treated missing values differently: generalized linear models use mean imputation to handle missing data. The missing values are replaced with the mean of the nonmissing values for that feature. Gradient boosting machines learn optimal splits in the decision trees for missing values. These algorithms do not impute missing data; instead, they find the best path in the decision trees for the observations with missing values. Deep learning algorithms perform mean imputation by default for handling missing data; they replace the missing values with the mean of the nonmissing values for that feature during training. Machine learning packages used for modeling also reject unimportant features, and this functionality was retained during modeling.

### Statistical Analyses: Model Development

The primary model was designed using a temporal reference point of patient arrival to the procedure room and thus only used data available before this event. The analysis was performed in R statistical software (R Foundation for Statistical Computing), using the *H2O* package of machine learning algorithms [29]. The models were generated and run via a server with a 2.9 GHz Intel processor with 96 GB RAM and a 64-bit operating system. This is comparable to a standard hospital computer system. Categorical variables were automatically processed to one-hot encoding. Predicted anesthesia CPT codes were used to characterize procedure types, using a previously published prediction model [21]. Multiple supervised machine learning regression algorithms were trained, including deep learning, gradient boosting machine, and stacked ensemble methods. In brief, deep learning helps to identify complex patterns, in which layers of nodes receive input and offer output, with successive layers representing more complex combinations of prior simpler layers [30]. By contrast, gradient boosting machines use weaker learners, specifically decision trees, by iteratively modifying the weights of each observation and progressively combining the trees together to improve the fit of the model [31]. Finally, stacked ensemble methods use combinations of strong learners (ie, deep learning, gradient boosting, and logistic regression) to optimize performance [32,33]. The best-performing model was further tuned, depending on the available hyperparameters for tuning. Hyperparameter tuning was accomplished using grid search, the default within the *H2O* package. Although the gradient boosting machine model was trained and tuned separately (*h2o.gbm* function in the *H2O* package), the deep learning and stacked ensemble models were generated using an automatic machine learning method (*autoML* function in the *H2O* package), which created and compared 10 distinct machine learning models.

### Data Partitioning

**Figure 1:**
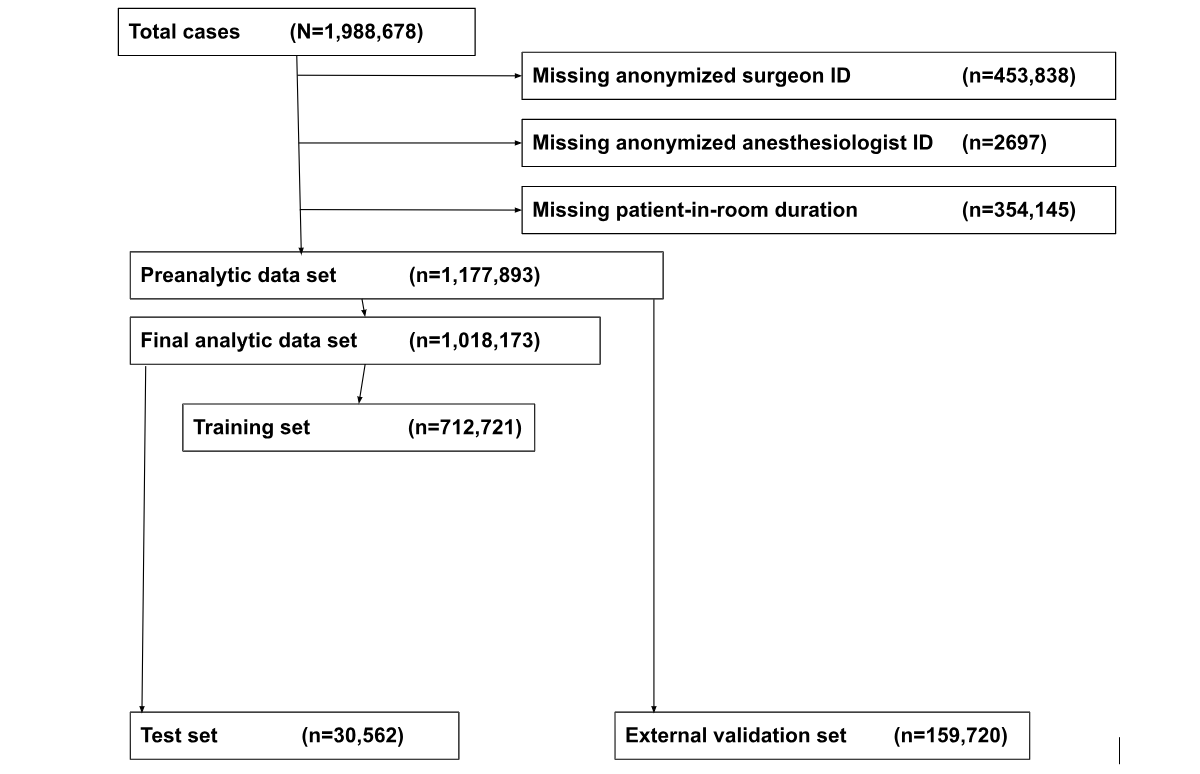
Study inclusion and exclusion criteria and machine learning model training and validation and testing schematic.

Split-set validation was used, in which 70% of the data were used for training and 30% for testing. Internal validation was additionally performed by using 5-fold cross-validation on the training set. In k-fold cross-validation, the training data set is divided into k subsets or *folds*. Each fold acts as a validation set for a specific model, whereas the remaining k-1 folds are used to train the model. This process is repeated k times, with each fold being used as a validation set exactly once. The model performance is then averaged over all k iterations. Data from 1 randomly selected institution were not included in the training or test sets and were used as a true *holdout* data set for external validation to further assess model generalizability (Figure 1).

### Performance Metrics

Model performance was assessed primarily using mean absolute error (MAE) and root mean square error (RMSE). In addition, for comparison with other published models and to further account for both distribution and outliers, median absolute error with IQR and mean absolute percentage error (MAPE) were also calculated. Because of high dimensional distributions and assumptions required for the generation of prediction intervals, two methods of assessing procedure duration variance were used: (1) a second model was trained, using the absolute error of each procedure prediction in the training set as the target output, and this model was then applied to the test set to generate a prediction error for each test set case; and (2) the loss function was modified to a quantile distribution, and 2 additional models were trained at values of 0.2 and 0.8 quantiles. A bootstrap method with 1000 repetitions was also attempted for generating a prediction interval, although we anticipated and confirmed that this was computationally expensive and time consuming beyond the utility of the workflow necessities of this algorithm.

The tuned best-performing final machine learning model was compared with a common historical reference model: historical procedure time by surgeon as the sole feature (independent variable) of a linear regression model. This was the same derived feature included in the machine learning models [5,34]. This feature was selected for comparison because *historical procedure time by surgeon* is commonly used by many institutions as the sole variable in their prediction models when cases are booked into the procedure room schedule. A comparison was performed using the Wilcoxon rank sum test on the model errors. Other available approaches such as Bayesian methods were not used for comparison owing to differences in the intended implementation, the availability of certain factors such as surgeon-estimated operative times, and the requirement to significantly modify the data structure.

All models were assessed for the distribution of error, overage (how frequently actual duration exceeded predicted duration), underage (how frequently actual duration underestimated predicted duration), and the percentage of procedures in which the predicted duration fell within 20% of the actual duration. Overage and underage are useful for broadly understanding whether the models tend to overestimate or underestimate the prediction. For each generated prediction interval (either predicted error or quantile loss function models), the percentage of procedures within the predicted range is also included as a performance metric. As performance metrics for procedure duration calculation vary widely in the literature and are often challenging to interpret by practicing clinicians and procedure room managers, we surveyed several procedure room managers to determine the most intuitive and useful metrics for use in a real-world setting. Finally, model use times were assessed to confirm that high-performing models are not too computationally intensive for practical use.

### Model Explainability Subanalysis

To facilitate improved explainability for applicable models, global and local plots of Shapley additive explanations (SHAP) values were developed [35]. SHAP is a framework built on game theory that provides greater interpretability of machine learning models. Global visualizations included permutation feature importance and SHAP global summary dot plots [36]. SHAP global summary dot plots relate the value of features to the outcome, as opposed to permutation feature importance, which relates the value of the feature to a selected performance metric. The SHAP value indicates how the value of a feature for a given procedure contributed to the prediction. A positive SHAP value contribution indicates that a feature increased the prediction above the average value, whereas a negative SHAP value contribution indicates that a feature decreased the prediction below the average value.

In addition, sample outputs were developed, including predicted duration, prediction interval, and SHAP local plots indicating the features, including direction and magnitude, that affected the output most for a given procedure. Similar approaches for explainability have been used in other medical machine learning applications within health care [37,38].

### Sensitivity Analyses

To better characterize the trade-offs between prediction model actionability and accuracy, 2 additional models were generated for use at different time points. One model used features restricted to those available at the time of surgical scheduling, and the other model used features expanded to those available after patient arrival to the procedure room, up to the time of procedure start (eg, surgical incision for operative procedures). Table 1 describes the models that were developed and the features that were determined available for use in the models.

To characterize the extent to which longer procedures influenced the results, 2 secondary subgroup analyses were performed, restricted to procedures lasting <180 minutes and <120 minutes. These 2 subgroup analyses were selected as clinically practical choices from the perspective of procedure room scheduling administrators. Given that longer procedures would likely be associated with greater error in prediction, this would provide an indication of the performance of shorter procedures.

## Results

### Population Baseline Characteristics

The training and testing data set included 1,018,173 unique procedures across 13 institutions, and the holdout data set included 159,720 procedures from a single institution (Figure 1). The number of cases at each deidentified center is provided in Table S2 in Multimedia Appendix 1. The median procedure duration was 94 (IQR 50-167) minutes; the 5th and 95th percentile durations were 21 and 361 minutes, respectively. Study population baseline characteristics, summarizing all features included in the models, are available in Table S3 in Multimedia Appendix 1. Creatinine, albumin, and international normalized ratio levels exceeded the 40% missing data threshold and were not included in further analyses.

### Primary Model Performance Metrics

After modeling and hyperparameter tuning, both the stacked ensemble model and the gradient boosting machine model resulted in comparable performance, with MAEs of 33 minutes and 34 minutes, respectively. The deep learning model demonstrated an MAE of 35 minutes and an RMSE of 57 minutes at the time of patient arrival to the procedure room, an MAE of 69 minutes and an RMSE of 85 minutes at the time of scheduling, and an MAE of 38 minutes and an RMSE of 62 minutes at the time of incision. The gradient boosting model was selected as the final model because the other performance metrics were comparable, and the tree-based nature of the algorithm allowed for global and local explainability. The final hyperparameters were 500 trees, maximum depth of 5, learning rate of 0.1, stopping tolerance of 0.01, and stopping metric of MAE, with all other hyperparameters at the default setting. The MAE was 19 (IQR 7.5-43) minutes, and the MAPE was 34%. The final model was applied to the single holdout institution for external validation, and model performance metrics are described in Table 2, including an MAE of 38 minutes, which is comparable with the MAE of the test set. For comparison, the performance metrics and specifications of the stacked ensemble model are provided in Tables S4 and S5, respectively, in Multimedia Appendix 1. The linear regression method using historical procedure time as the sole feature (independent variable) demonstrated an MAE of 43 minutes on the test set and an MAE of 48 minutes on the external validation set and an MAPE of 45%. The difference in error between the linear regression model and the final machine learning model was statistically significant (*P*<.001).

**Figure 2:**
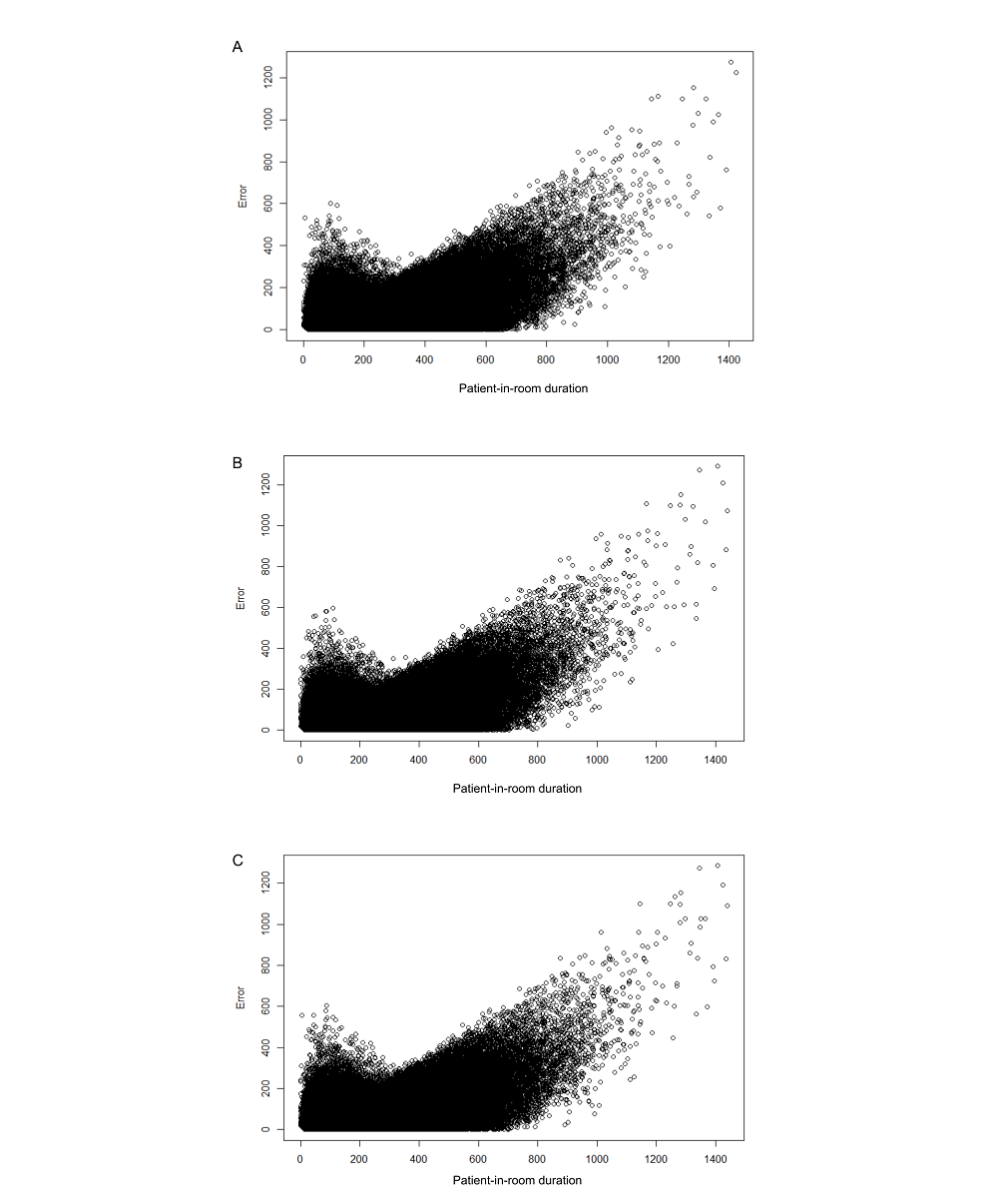
Patient-in-room duration plotted against prediction error. (A) Time of Patient in OR [Operating Room] model (primary model). (B) Time of Scheduling model (secondary model). (C) Time of Surgical Incision model (secondary model).

Using 2 different methods for generating prediction intervals, it was determined that the error prediction model resulted in actual procedure times within the predicted range 64% of the time within the primary analysis (Table 2). As anticipated, the bootstrap method was highly computationally expensive (at least 15 min to compute a single prediction interval) and considered impractical for the workflow setting. The prediction intervals of longer-duration procedures were wider than shorter-duration procedures. From observation of the error distribution plots (Figure 2), it seemed clear that longer procedures typically tended to have greater error than shorter procedures. The computation time to predict on the test set (>300,000 cases) was 10 seconds.

**Table 2:** Performance of optimized surgical duration prediction models at each time point: test set, external validation set, and prediction intervals.

		*Time of Scheduling* model (secondary model)	*Time of Patient in OR*^a^ model (primary model)	*Time of Surgical Incision* model (secondary model)
**Test set**				
	Mean absolute error (min), mean (SD)	34 (47)	34 (47)	34 (47)
	Root mean square error, min	59	59	59
	Overage, %	58	58	58
	Underage, %	42	42	42
	Prediction within 20% of actual duration, %	46	46	46
**External validation set**				
	Mean absolute error (min), mean (SD)	38 (52)	38 (52)	38 (52)
**Prediction intervals**				
	Actual duration within prediction interval, %	Error prediction model: 64; quantile loss function: 61	Error prediction model: 63; quantile loss function: 61	Error prediction model: 65; quantile loss function: 61

^a^OR: operating room.

### SHAP Global Summary and Feature Importance

**Figure 3:**
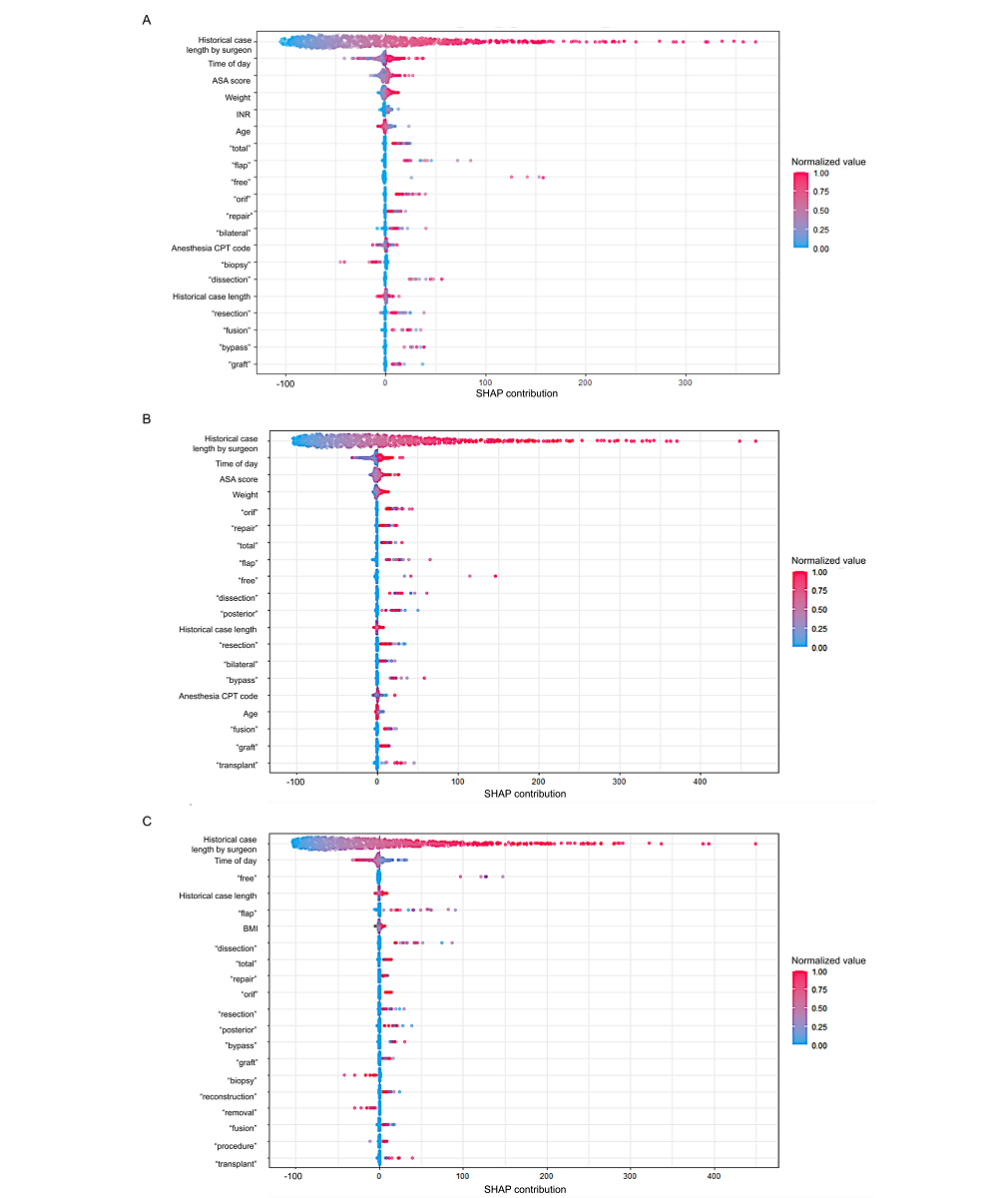
Shapley additive explanations (SHAP) global summary dot plots. (A) Time of Patient in OR [Operating Room] model (primary model). (B) Time of Scheduling model (secondary model). (C) Time of Surgical Incision model (secondary model). The feature ranking (y-axis) implies the order of importance of the feature. The SHAP value (x-axis) is a unified index reflecting the impact of a feature on the model output. In each feature importance row, the attributions of all cases to the outcome were plotted using different colored dots, of which the redder dots represent a higher (or positive, if binary) value, and the bluer dots represent a low (or negative, if binary) value, along a gradient from red to blue. ASA: American Society of Anesthesiologists; CPT: current procedural terminology; INR: international normalized ratio.

The features with the highest importance by feature importance were historical procedure duration by surgeon, the word “free” in the procedure text (eg, “free flap”), and the time of day. The features with the highest importance based on global SHAP values were historical procedure duration by surgeon, the time of day, and American Society of Anesthesiologists physical status score. SHAP global summary dot plots of each time point model are shown in Figure 3. Permutation feature importance for each time point model is shown in Figure S1 in Multimedia Appendix 1.

### Sample Output

**Figure 4:**
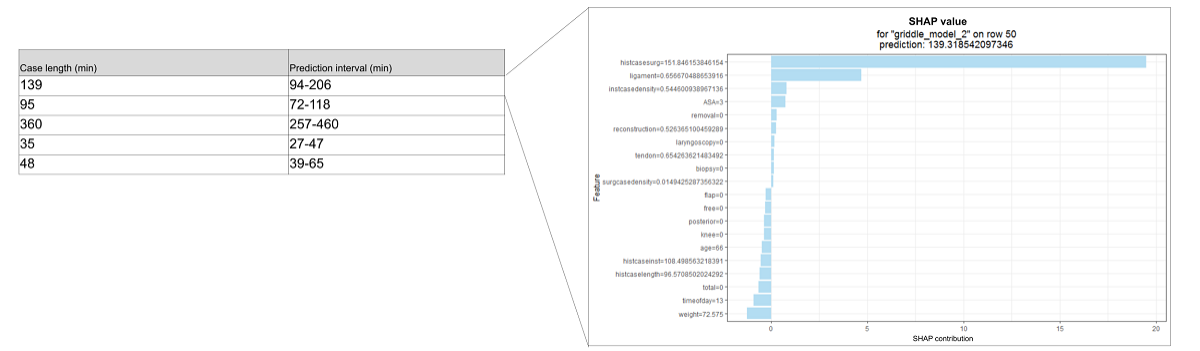
Sample output, including Shapley additive explanations (SHAP) local plot. A positive SHAP value contribution indicates that a feature increased the prediction above the average value, whereas a negative SHAP value contribution indicates that a feature decreased the prediction below the average value.

Model outputs feasible for use in real time included predicted time in minutes, the prediction interval as a range, and the SHAP local explainability plot. As examples, outputs of 5 randomly selected procedures from the test set are shown in Figure 4. For further explanation, a local explainability plot can be easily generated as shown.

### Sensitivity Analyses

In a sensitivity analysis restricted to features available at the time of procedure scheduling, the final model was the gradient boosting machine with an MAE of 34 minutes; for the analysis expanding features to those available up to the time of procedure start (eg, surgical incision), the final model was again the gradient boosting machine with an MAE of 34 minutes (Table 2). At each time point, unused or unimportant columns were dropped by the machine learning algorithm (Table S6 in Multimedia Appendix 1). In the secondary subgroup analyses restricted to shorter procedures, when applying the primary model to procedures lasting <180 minutes, the MAE was 24 minutes, and for procedures lasting <120 minutes, the MAE was 22 minutes.

## Discussion

### Principal Findings

In this study, we generated machine learning models for the prediction of procedure duration. The final model was the gradient boosting machine, with an MAE of 34 minutes in the test set and an MAE of 38 minutes in the external validation set. This multicenter data set provided a high procedure volume and a wide breadth of procedure types across multiple institutions. Model output included a prediction interval and local explainability for each prediction.

The features with the highest permutation importance were historical procedure duration by surgeon, the word “free” within the procedure text, and the time of day, and those with the highest SHAP values were historical procedure duration by surgeon, American Society of Anesthesiologists physical status score, and the time of day. We speculate that the word “free” having high permutation importance is related to the nature of “free flap” surgery, historically a lengthy procedure. The nonlinear interactions among procedure, surgeon, patient illness severity, and resource availability (the time of day) describe the largest component of the prediction of our model.

Prediction plots suggest that error increases with procedure duration. This result is corroborated by the sensitivity analysis that examined only procedures lasting <120 minutes and <180 minutes, both of which resulted in a lower MAE. Future work might explore different models for different ranges of booking duration because the models might identify factors in longer procedures that are different from those in shorter procedures.

### Supporting Literature

This study expands beyond previous work on single surgical specialties or single-center studies [6,10,13]. Our results show strong performance similarly improved on historical prediction methods [26]. Although it is difficult to compare across different data sets, our model performed grossly better than a single-center model for the prediction of robotic surgery duration (RMSE 80.2 min) [10] and a prospectively evaluated single-center model (MAE 49.5 min) [39]. Compared with a proprietary model tested on 1000 procedures at a single center, which demonstrated a median absolute error of 20 (IQR 10-28) minutes, our model performed comparably with a median absolute error of 19 (IQR 7.5-43) minutes [13]. When compared with a single-center model with a similar analytic approach, our model fared slightly poorer, with an MAPE of 34%, compared with the best surgeon-specific model, with an MAPE of 27% [26]. The approach included multiple surgeon-specific models [26] (as opposed to our unified model, which included all surgeons); considering the high importance of historical case time by surgeon within our model, this difference in performance is expected*.*

### Study Strengths

There have been successful attempts at predicting procedure length, although implementation is often limited by a number of factors, including moderate performance, cumbersome workflow, or the high frequency of unavailable variables. Our major strength is the vast amount of multicenter data. The inherent heterogeneity of practice environments permits potential broader generalizability and customizability of the model, as evidenced by the performance on the test sets and external validation sets. In addition, our approach used commonly available data within the electronic health record that does not rely on human input (ie, human-estimated procedure times), permitting potential improved external implementation.

Our study also introduces several derived features that can be used in other similar projects because the explainability analyses suggest that *historical case length by surgeon*, *institution case density*, *historical case length by institution*, and *historical case length* all have an impact on performance. These features are relatively simple to compute using the code provided in Multimedia Appendix 1 (refer to Supplementary Code subsection).

Our aim was to develop an easy-to-implement solution with an easy-to-interpret and valuable output. In addition to procedure duration prediction, prediction interval as a measure of variance is useful. Procedures with a high variance can be viewed as less predictable and scheduled accordingly, either after more predictable duration procedures or with no procedures to follow. Furthermore, including an explainability aims to minimize *black box* modeling, building algorithm trust and allowing valuable insight. The global SHAP summary plots improve upon variable importance by relating features to outcome as opposed to relating features to performance metric. The local SHAP plots offer explanations of the drivers of an individual prediction. Although most of these features may not be modifiable, this provides users a data-driven understanding of the drivers of procedure duration [39]; for example, case durations may be *overread* by a procedure room clinician administrator (similar to an electrocardiogram being overread by a cardiologist), and they may be better able to trust or not trust a predicted procedure duration, based on what is most influencing the prediction, and make modifications to procedure room schedule and staffing accordingly. Ideally, the algorithms (similar to most health care artificial intelligence [AI] applications) are used in conjunction with expert opinion and not typically as a sole arbiter of decision-making. In addition, actual cases may on occasion deviate from the booked case. Using the provided example, for instance, the surgeon may decide immediately before the procedure that they will now perform a free flap (or not perform one), or the time of day changes owing to an urgent add-on case bumping the current case. Through a quick review of the explainability, the procedure room managers can estimate how this may affect the case duration and plan case allocation and staffing accordingly for the procedures to follow. Finally, there is currently a systemic lack of trust in health care AI applications, as evidenced by several thought leaders in AI, medical ethics, and medical law [40-42]. To a significant degree, this is a result of the black box nature of most health care AI applications, seeding distrust for most health care clinicians. Providing explainability allows far greater transparency in the decision-making process and is supported by several prior studies [43-47].

### Unexpected Findings

Performance metrics at each time point were ultimately similar, and many of the additional features available at later time points were dropped by the machine learning algorithm for being unimportant to model prediction. This suggests that the information provided by many of these features does not provide an overall improvement in the performance of the models and that the features with the highest importance also tend to be the ones with greater availability and at earlier time points. This provides reassurance that the model is likely to be robust within various data schemas as long as the natural language processing and feature engineering remain consistent and use electronic health record features routinely available at a majority of institutions. In addition, this can be useful for case schedulers to fill a procedure room block efficiently, and procedure room managers can appropriately allocate resources potentially earlier.

### Limitations

Despite the performance of the models, there are still a number of limitations to our approach. First, although the volume of data is high, the data as provided are relatively uniformly curated. Although this may be seen as a benefit from a data analysis perspective, it does mean that the precise data processing performed here is specific to this data structure and not necessarily to local institution data structure. The single-institution validation model aids in supporting potential generalizability, but data processing may differ by institution. Two simple solutions include using a shadow copy of local data that restructures data to the same schema or retraining of the model using local data schema. Second, the features with the highest variable importance need to be both available and reliable. Third, financial analyses related to time are beyond the scope of this study owing to multiple factors being involved, including staffing models and staffing ratios, procedure type, procedure acuity, payer status, and local policy [48,49]. Next, there may be procedures that occurred on the same patient. Ultimately, the explainability analyses suggested that patient characteristics had little contribution to make to the model performance compared with the more impactful derived features and natural language processed procedure text. In addition, the data used in this study are all from before the international COVID-19 pandemic because that is when the analysis was initially performed. The algorithm would have to be updated to include more recent postpandemic data because hospital systems are likely to have changed. Finally, all institutions in this data set are from the United States, which may limit international generalizability.

### Use in Practice

We are transparent in our design and have provided the code to implement the models in Multimedia Appendix 1. A code use schematic (Figure S2 in Multimedia Appendix 1) aids in understanding the relationship of processing data, updating models, and generating output. All code is available in a web-based repository [50]. First, we provide the trained models in R *H2O* format, which can be applied directly to new data (upcoming procedures) to generate predictions (Multimedia Appendix 1 [refer to R Code: Making Predictions Using Created Machine Learning Models]). We provide the code needed to preprocess data, including generating derived experience features and natural language processing of procedure text (Multimedia Appendix 1 [refer to R Code for Data Processing]). This preprocessing code can generate a new training set or can be applied to reformat new data for the provided models. Finally, we provide the code to generate new models or to update the existing models with more current data, including up-to-date derived experience features (Multimedia Appendix 1 [refer to R Code: Training and Testing ML Models]).

For use in practice, ideally, the model will be installed and maintained locally. It can be rebuilt periodically to avoid excessive computational requirements. Time for prediction is negligible (1 s −0.3 s to +0.3 s). The model can be used as the default prediction when scheduling cases, or, if used at the time of scheduling, it can drive alerts for procedures with scheduled times incongruent with predicted times. A recent example of a single-center prospective implementation of a similar model suggests that there is a benefit to using these methods with regard to accurate prediction of surgical times and impact on workflow [39]. However, ultimately, institutional policy will largely steer implementation; for example, many institutions do not routinely use a surgeon or scheduler estimate at procedure booking [51]. The use of this tool obviates the need for individualized input. Future studies are necessary to prospectively validate the performance of procedure duration prediction models integrated into daily workflow for clinician and administrator use in real time.

### Conclusions

We report a robust and generalizable model for the prediction of procedure duration and variability within an acceptable tolerance derived from rigorous testing of machine learning models applied to a large multicenter data set. Our findings may guide the future development of procedure room workflow implementation of procedure duration prediction models.

## Appendix

Multimedia Appendix 1 Supplementary tables and figures, as well as R code for data manipulation, model training, and model building.

## Abbreviations

AI: artificial intelligence
CPT: current procedural terminology
MAE: mean absolute error
MAPE: mean absolute percentage error
MPOG: Multicenter Perioperative Outcomes Group
NYU: New York University
RMSE: root mean square error
SHAP: Shapley additive explanations
