# Supplementary material for: Machine Learning for the Prediction of Procedural Case Durations Developed Using a Large Multicenter Database: Algorithm Development and Validation Study
Source: JMIR AI. 2023 Sep 8;2:e44909. doi: 10.2196/44909 (PMC11041482; doi:10.2196/44909)
Supplement: Multimedia Appendix 1 [file ai_v2i1e44909_app1.docx]

**SUPPLEMENT**

**Explainable Machine Learning for Prediction of Procedural Case Durations Developed Using a Large Multicenter Database: Algorithm Development and Validation**

[**Supplementary Tables 2**](#_xb7u71221f3m)

[Table S1. List of health care centers included in data set 2](#_bv81rjghg1v7)

[Table S2. Number of cases at each included health care center 3](#_iecs5alvzz0i)

[Table S3. Study Population Baseline Characteristics of Full Analytic Dataset, including frequency of missing data for each feature 4](#_snzpjjl9rde1)

[Table S4. Performance Metrics of Stacked Ensemble Model at Each Time Point 10](#_15qmqrul7olb)

[Table S5. Stacked Ensemble Model Specifications (including submodels and hyperparameters) 11](#_4vmeubnnoig)

[Table S6. Columns (features) dropped by machine learning algorithm at each time point 12](#_vxl8sud4w785)

[**Supplementary Figures 13**](#_w77b934r4snj)

[Figure S1. Permutation feature importance of features included in final machine learning model at each time point, test set. a) Time of Patient in OR, primary model; b) Time of scheduling, secondary model; c) Time of Surgical Incision, secondary model 13](#_xkgleg3auu72)

[Figure S2. Code Use Schematic 14](#_h9ab6muwvj5f)

[**Supplementary Code 15**](#_yepxsqo0xe5u)

[C1. R Code for Data Processing 15](#_hpnuxtvnoz3y)

[C2. R Code: Training and Testing ML Models 21](#_njhohtbosxg7)

[C3. R Code: Making Predictions Using Created Machine Learning Models 26](#_d1x5kk1g5y7k)

#

# **Supplementary Tables**

## *Table S1. List of health care centers included in data set*

| Health Care Center (alphabetical order) |
| --- |
| Dartmouth-Hitchcock Medical Center |
| Holland Hospital |
| Memorial Sloan Kettering Cancer Center |
| Stanford Hospitals and Clinics |
| Trinity - Mercy Health Muskegon |
| Trinity - St. Joseph Mercy Ann Arbor |
| Trinity - St. Joseph Oakland |
| Trinity - St. Mary Mercy Livonia |
| University of California San Francisco |
| University of Michigan Health - Ann Arbor |
| University of Oklahoma Health Sciences Center |
| University of Tennessee Medical Center |
| University of Washington Medical Center |
| Washington University School of Medicine |

## *Table S2. Number of cases at each included health care center*

| Deidentified Institution | Case Count |
| --- | --- |
| 3 | 316786 |
| 8 | 101984 |
| 15 | 62018 |
| 18 | 39268 |
| 19 | 98429 |
| 20 | 174535 |
| 21 | 259816 |
| 24 | 109313 |
| 25 | 163498 |
| 30 | 171857 |
| 31 | 115570 |
| 46 | 52230 |
| 48 | 157668 |
| 52 | 165706 |

##

## *Table S3. Study Population Baseline Characteristics of Full Analytic Dataset, including frequency of missing data for each feature*

| Feature | N | % | Missing |
| --- | --- | --- | --- |
| Admit Type |  |  |  |
| 23 hour observation | 26586 | 2.61 |  |
| Admit | 167820 | 16.48 |  |
| Emergency | 3863 | 0.38 |  |
| Inpatient | 266980 | 26.22 |  |
| Other | 1425 | 0.14 |  |
| Outpatient | 532336 | 52.28 |  |
| Unknown admission type | 19011 | 1.87 |  |
| Unknown concept | 152 | 0.01 |  |
| Sex |  |  |  |
| Female | 529503 | 52.01 |  |
| Male | 488387 | 47.97 |  |
| Unknown | 283 | 0.03 |  |
| Age (years) | 54 (33-67) |  |  |
| Body Mass Index | 27 (23-32) |  | 81062 (8.0) |
| ASA Physical Status Score |  |  |  |
| 1 | 105207 | 10.33 |  |
| 2 | 425668 | 41.81 |  |
| 3 | 406864 | 39.96 |  |
| 4 | 74798 | 7.35 |  |
| 5 | 3299 | 0.32 |  |
| 6 | 193 | 0.02 |  |
| Unknown | 2144 | 0.21 |  |
| Baseline Mean Arterial Pressure | 91 (78-103) |  | 2795 (0.27) |
| Holiday | 2960 | 0.29 |  |
| Weekend | 38047 | 3.74 |  |
| Service |  |  |  |
| anesthesiology | 739 | 0.07 |  |
| burn | 1243 | 0.12 |  |
| cardiac | 21149 | 2.08 |  |
| cardiothoracic | 7719 | 0.76 |  |
| dentistry | 3945 | 0.39 |  |
| dermatology | 411 | 0.04 |  |
| general | 121455 | 11.93 |  |
| gynecology | 23622 | 2.32 |  |
| cardiology | 10972 | 1.08 |  |
| gastroenterology | 95454 | 9.38 |  |
| hematology/oncology | 1517 | 0.15 |  |
| medical - not specified | 229 | 0.02 |  |
| medical - other | 1870 | 0.18 |  |
| pulmonology | 3762 | 0.37 |  |
| neurology | 72 | 0.01 |  |
| neurosurgery | 47418 | 4.66 |  |
| obstetrics | 2288 | 0.22 |  |
| ob/gyn | 30223 | 2.97 |  |
| ophthalmology | 73521 | 7.22 |  |
| oral/maxillofacial | 9549 | 0.94 |  |
| orthopedics | 147796 | 14.52 |  |
| otolaryngology | 74247 | 7.29 |  |
| pain management | 8049 | 0.79 |  |
| pediatric cardiac surgery | 684 | 0.07 |  |
| pediatric cardiology | 817 | 0.08 |  |
| pediatric gastroenterology | 14 | 0.00 |  |
| pediatric general surgery | 1624 | 0.16 |  |
| pediatric hem/onc | 658 | 0.06 |  |
| pediatric neurosurgery | 323 | 0.03 |  |
| pediatric orthopedic surgery | 670 | 0.07 |  |
| pediatric surgery | 2367 | 0.23 |  |
| pediatric urology | 864 | 0.08 |  |
| plastics | 42782 | 4.20 |  |
| podiatry | 4030 | 0.40 |  |
| psychiatry | 6723 | 0.66 |  |
| radiation oncology | 854 | 0.08 |  |
| radiology - diagnostic | 32 | 0.00 |  |
| radiology - interventional | 6406 | 0.63 |  |
| radiology - unspecified | 10980 | 1.08 |  |
| surgery - oncology | 384 | 0.04 |  |
| service not specified | 10681 | 1.05 |  |
| other service | 461 | 0.05 |  |
| service not listed | 20110 | 1.98 |  |
| thoracic | 14502 | 1.42 |  |
| transplant | 8250 | 0.81 |  |
| trauma | 5450 | 0.54 |  |
| unknown concept | 100435 | 9.86 |  |
| urology | 67420 | 6.62 |  |
| vascular | 23179 | 2.28 |  |
| room type |  | 0.00 |  |
| Acute care hospital - intensive care unit procedure | 149 | 0.01 |  |
| Acute care hospital - minor procedure room | 48979 | 4.81 |  |
| Acute care hospital - mixed use operating room | 450161 | 44.21 |  |
| Acute care hospital - remote diagnostic radiology procedure room | 875 | 0.09 |  |
| Acute care hospital - remote interventional radiology procedure room | 18008 | 1.77 |  |
| Acute care hospital - remote minor procedure room | 48519 | 4.77 |  |
| Attached ambulatory surgery center - minor procedure room | 7128 | 0.70 |  |
| Attached ambulatory surgery center - outpatient operating room | 46037 | 4.52 |  |
| Freestanding ambulatory surgery center - minor procedure room | 677 | 0.07 |  |
| Freestanding ambulatory surgery center - outpatient operating room | 117845 | 11.57 |  |
| Freestanding ambulatory surgery center - remote minor procedure room | 7564 | 0.74 |  |
| Obstetrics - labor and delivery room | 8579 | 0.84 |  |
| Obstetrics - operating room | 5266 | 0.52 |  |
| Office based anesthesia operating room | 3 | 0.00 |  |
| Other procedure room type | 17441 | 1.71 |  |
| Pediatric acute care hospital - minor procedure room | 8101 | 0.80 |  |
| Pediatric acute care hospital - mixed use operating room | 64350 | 6.32 |  |
| Pediatric acute care hospital - remote diagnostic radiology procedure room | 3952 | 0.39 |  |
| Pediatric acute care hospital - remote interventional radiology procedure room | 2163 | 0.21 |  |
| Pediatric acute care hospital - remote minor procedure room | 2556 | 0.25 |  |
| Unknown Concept | 138591 | 13.61 |  |
| Unknown procedure room type | 21229 | 2.09 |  |
| Creatinine | 0.84 (0.69-1.03) |  | 406419 (40) |
| Hemoglobin | 12.9 (11.3-14.1) |  | 397565 (39.0) |
| Albumin | 4 (3.6-4.3) |  | 556771 (54.7) |
| INR | 1.1 (1.0-1.2) |  | 651485 (64) |
| CHF | 61979 | 6.09 | 82887 (8.1) |
| Arrythmia | 138249 | 13.58 | 82887 (8.1) |
| Valvular Dysfunction | 45622 | 4.48 | 82887 (8.1) |
| Peripheral Vascular Disease | 55511 | 5.45 | 82887 (8.1) |
| Hypertension with complications | 78275 | 7.69 | 82887 (8.1) |
| Hypertension without complications | 276799 | 27.19 | 82887 (8.1) |
| Diabetes with complications | 36944 | 3.63 | 82887 (8.1) |
| Diabetes without complications | 95755 | 9.40 | 82887 (8.1) |
| Pulmonary Disease | 125943 | 12.37 | 82887 (8.1) |
| Renal Disease | 80140 | 7.87 | 82887 (8.1) |
| Time of Day |  | 0.00 |  |
| 0 | 2004 | 0.20 |  |
| 1 | 1761 | 0.17 |  |
| 2 | 1598 | 0.16 |  |
| 3 | 1512 | 0.15 |  |
| 4 | 1464 | 0.14 |  |
| 5 | 1668 | 0.16 |  |
| 6 | 11302 | 1.11 |  |
| 7 | 198474 | 19.49 |  |
| 8 | 121523 | 11.94 |  |
| 9 | 96393 | 9.47 |  |
| 10 | 104071 | 10.22 |  |
| 11 | 97115 | 9.54 |  |
| 12 | 94956 | 9.33 |  |
| 13 | 88560 | 8.70 |  |
| 14 | 71219 | 6.99 |  |
| 15 | 50126 | 4.92 |  |
| 16 | 30016 | 2.95 |  |
| 17 | 15941 | 1.57 |  |
| 18 | 8802 | 0.86 |  |
| 19 | 5749 | 0.56 |  |
| 20 | 4878 | 0.48 |  |
| 21 | 3790 | 0.37 |  |
| 22 | 2947 | 0.29 |  |
| 23 | 2304 | 0.23 |  |
| Arterial line | 132569 | 13.02 | 4151 (0.04) |
| Block |  |  |  |
| Adductor Canal | 1846 | 0.18 |  |
| Ankle | 494 | 0.05 |  |
| Axillary | 636 | 0.06 |  |
| Bier | 3716 | 0.36 |  |
| Multiple | 9500 | 0.93 |  |
| Unknown | 29585 | 2.91 |  |
| Caudal | 1092 | 0.11 |  |
| Cervical Plexus | 379 | 0.04 |  |
| Eye block | 2744 | 0.27 |  |
| Femoral | 864 | 0.08 |  |
| Infraclavicular | 1227 | 0.12 |  |
| Inguinal | 1 | 0.00 |  |
| Interscalene | 3883 | 0.38 |  |
| Lumbar Plexus | 1 | 0.00 |  |
| None | 949623 | 93.27 |  |
| Perineural | 5185 | 0.51 |  |
| Popliteal | 756 | 0.07 |  |
| Saphenous | 134 | 0.01 |  |
| Sciatic | 488 | 0.05 |  |
| Supraclavicular | 4366 | 0.43 |  |
| TAP | 1653 | 0.16 |  |
| Epidural | 33874 | 3.33 |  |
| Anesthesia Type |  | 0.00 |  |
| Both ETT and LMA | 9149 | 0.90 |  |
| ETT | 438129 | 43.03 |  |
| Inhaled only | 11023 | 1.08 |  |
| LMA | 153145 | 15.04 |  |
| NMB only | 8307 | 0.82 |  |
| Unknown | 108818 | 10.69 |  |
| No | 289602 | 28.44 |  |
| Neuraxial |  |  |  |
| Caudal | 2205 | 0.22 |  |
| CSE | 11218 | 1.10 |  |
| Epidural | 20252 | 1.99 |  |
| Multiple | 2394 | 0.24 |  |
| Unknown | 23402 | 2.30 |  |
| None | 950055 | 93.31 |  |
| Spinal | 8647 | 0.85 |  |

## *Table S4. Performance Metrics of Stacked Ensemble Model at Each Time Point*

|  | “Time of Scheduling” Model (Secondary Model) | “Time of Patient in OR” Model (Primary Model) | “Time of Surgical Incision” Model  (Secondary Model) |
| --- | --- | --- | --- |
| Mean Absolute Error (SD), minutes | 33 (56) | 33 (56) | 33 (56) |
| Root Mean Squared Error ,minutes | 56 | 57 | 57 |
| Overage, % | 57 | 57 | 57 |
| Underage, % | 43 | 43 | 43 |
| Prediction within 20% of actual duration, % | 50 | 50 | 50 |
| R squared | 0.766 | 0.766 | 0.766 |
|  | External Validation Set | | |
| Mean Absolute Error (minutes) | 40 | 40 | 40 |
|  | Error Prediction Model | | |
| Actual duration within prediction interval (%) | 52 | 52 | 52 |

## *Table S5. Stacked Ensemble Model Specifications (including submodels and hyperparameters)*

“Time of Patient in OR” Model (Primary Model)


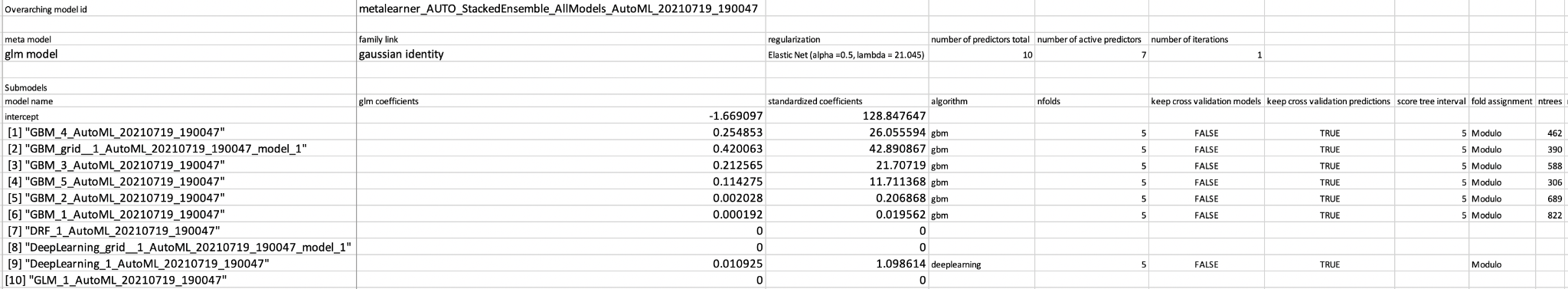


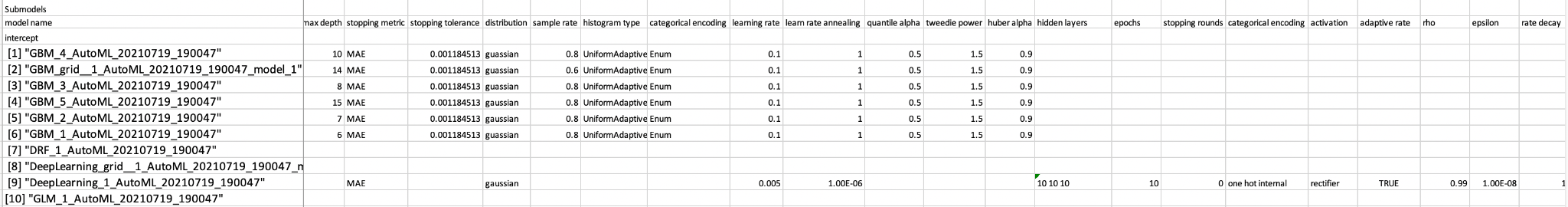


“Time of Scheduling” Model (Secondary Model)


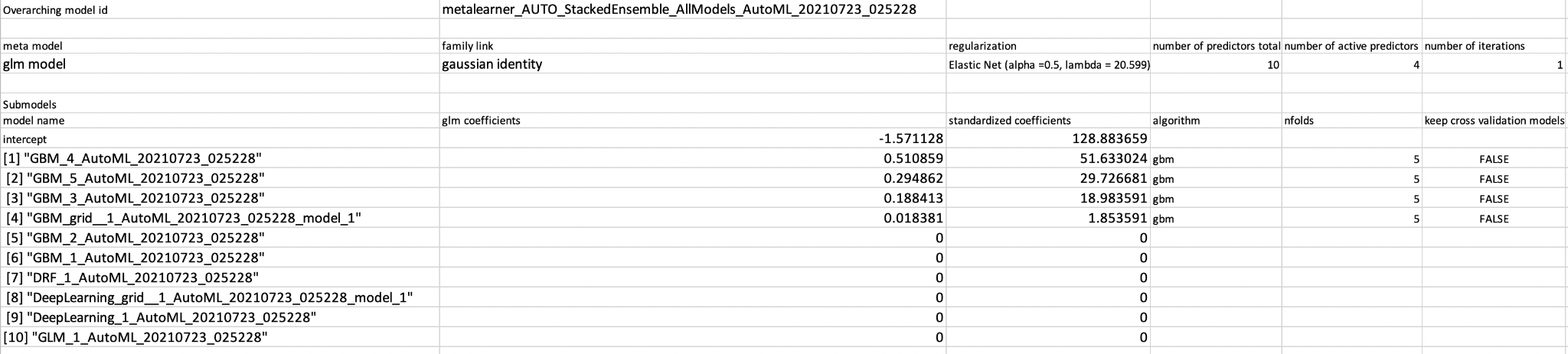


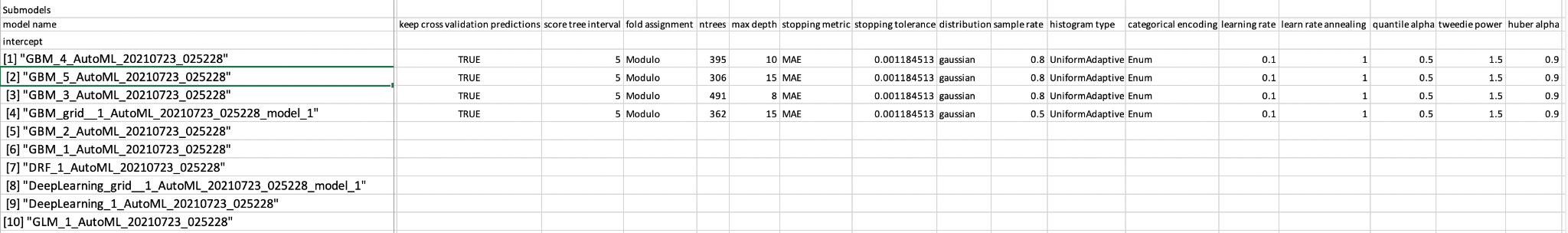


“Time of Surgical Incision” Model (Secondary Model)


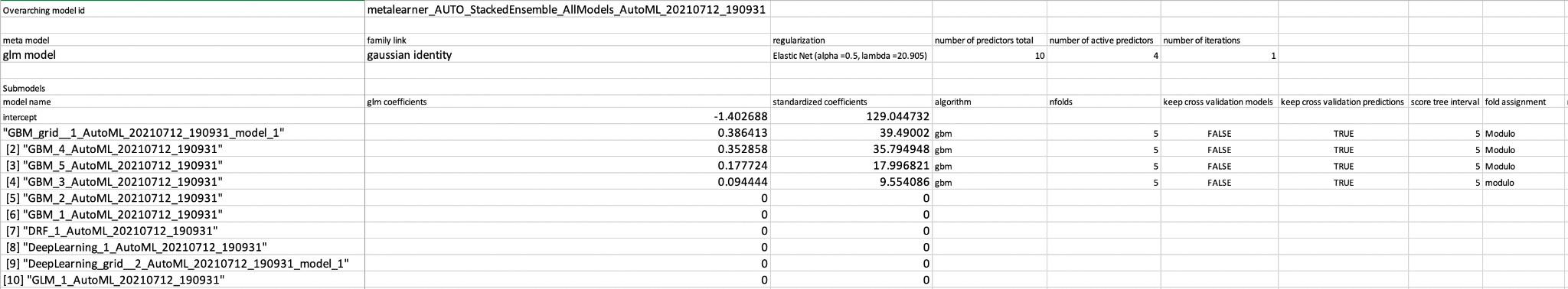


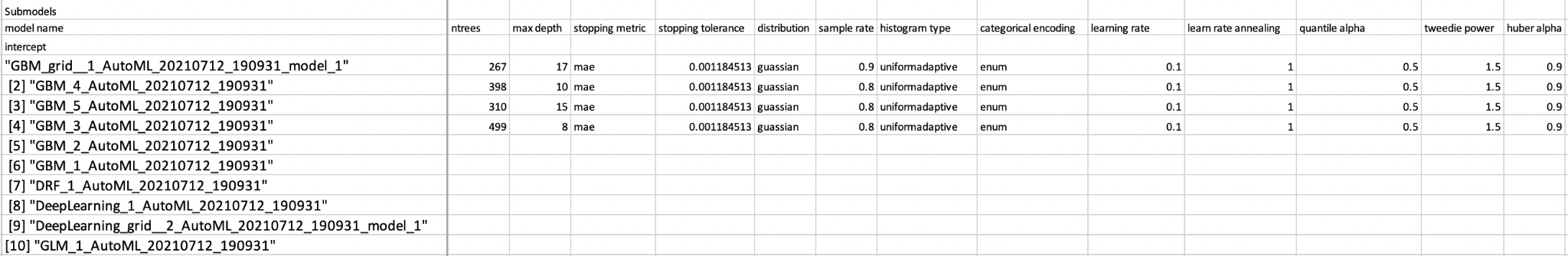


## *Table S6. Columns (features) dropped by machine learning algorithm at each time point*

| **Model** | **Dropped columns** |
| --- | --- |
| “Time of Patient in OR” Model (Primary Model) | Congestive heart failure, General anesthesia used, sex, uncomplicated diabetes, complicated hypertension, baseline mean arterial pressure, emergency, complicated diabetes, room type, uncomplicated hypertension, renal, surgeon identity, pulmonary disease, surgical service, arrhythmia, valvular disease, admit type, cardiac surgery, peripheral vascular disease |
| “Time of Scheduling” Model (Secondary Model) | Congestive heart failure, sex, uncomplicated diabetes, complicated hypertension, complicated diabetes, room type, uncomplicated hypertension, renal, surgeon identity, pulmonary disease, surgical service, arrhythmia, valvular disease, admit type, cardiac surgery, peripheral vascular disease |
| “Time of Surgical Incision” Model  (Secondary Model) | Congestive heart failure, lma, sex, complicated hypertension, baseline mean arterial pressure, complicated diabetes, anestime, artline, surgeon identity, block, genanes, pvd, uncomplicated diabetes, room type, uncomplicated hypertension, renal, neurax, pulmonary disease, service, arrhythmia, valvular disease, admit type, cardiac surgery |

# **Supplementary Figures**

## *Figure S1. Permutation feature importance of features included in final machine learning model at each time point, test set. a) Time of Patient in OR, primary model; b) Time of scheduling, secondary model; c) Time of Surgical Incision, secondary model*


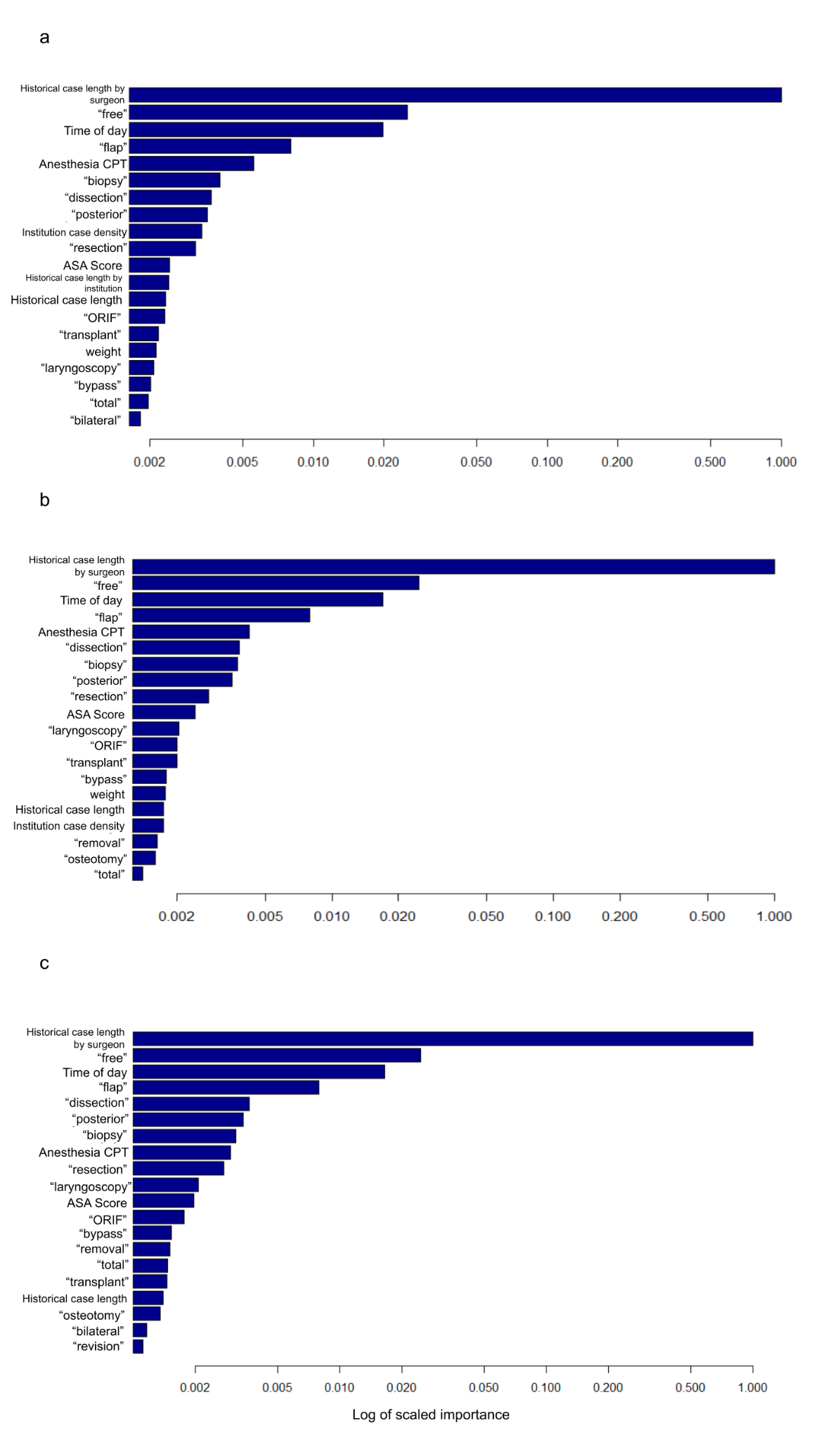


## *Figure S2. Code Use Schematic*


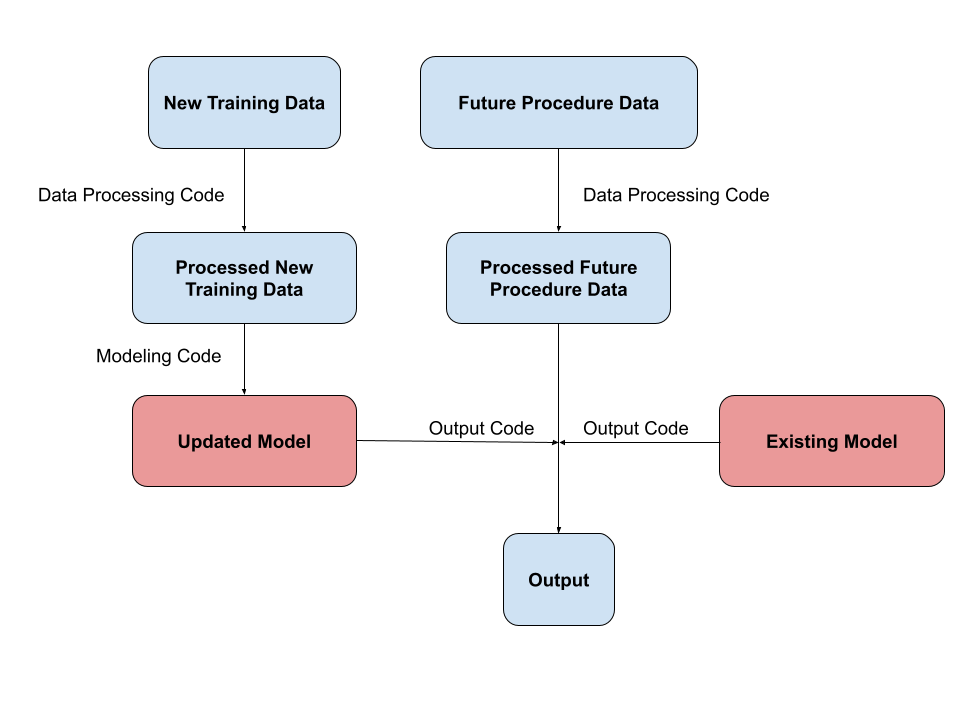


# **Supplementary Code**

(All code available at http://github.com/skendalemd/SuperProcLengthEngine)

## **C1. R Code for Data Processing**

library(dplyr)

library(tidyr)

library(rJava)

library(RWeka)

library(tm)

library(caret)

library(lubridate)

library(mice)

library(RODBC) #database connection

###########

#Read Data#

###########

#read provider data (the example below uses a database connection through SQL using the RODBC library)

options(stringsAsFactors = FALSE)

cn <-odbcDriverConnect(connection=...;server=...;database=...;trusted_connection=yes;")

provs <- sqlQuery(cn,"select * from ...")

names(provs)[1]<-"MPOG_Case_ID"

#get primary surgeon id

getsurgid<-provs %>%

filter(MPOG_Staff_Role_Concept_ID==6006) %>%

group_by(MPOG_Case_ID) %>%

slice(1) %>%

rename(surgid=AIMS_Staff_ID)

#get primary anes id

getanesid<-provs %>%

filter(MPOG_Staff_Role_Concept_ID==6000) %>%

group_by(MPOG_Case_ID) %>%

slice(1) %>%

rename(anesid=AIMS_Staff_ID)

#read cases (the example below reads data from a CSV)

setwd("C:/...") #set your working directory to the location of your data

cases<-read.csv(“your_case_data”)

names(cases)[1]<-"MPOG_Case_ID"

#add surgeon and anes id to cases

cases<-merge(cases,getsurgid,by="MPOG_Case_ID")

cases<-merge(cases,getanesid,by="MPOG_Case_ID")

cases$Date<-as.Date(cases$Anesthesia.Start)

#separate Anesthesia CPTs into separate columns

#Anesthesia CPT generated by prediction model

cases<-cases %>% separate(Predicted.Anes.CPTs, c("CPT1","CPT2","CPT3"),extra="drop")

#convert duration variable to numeric format

cases$Patient.In.Room.Duration<-as.numeric(as.character(cases$Patient.In.Room.Duration))

#exclude case length NA, negative, or greater than 1440 minutes

cases<-cases %>%

filter(!is.na(Patient.In.Room.Duration)) %>%

filter(Patient.In.Room.Duration>0) %>%

filter(Patient.In.Room.Duration<1440)

#################################################################

####Below code is for monthly engineered experience features

#################################################################

#--Below code is only for monthly engineered experience features

firstdate=min(cases$Date)

lastdate=max(cases$Date)

rangebymonth=seq(firstdate,lastdate,by="month")

cases<-cases %>%

mutate(surgtotexp=0) %>%

mutate(surgcaseexp=0) %>%

mutate(instcaseexp=0) %>%

mutate(histcaselength=0) %>%

mutate(histcaseinst=0) %>%

mutate(histcasesurg=0) %>%

mutate(surgtotdensity=0) %>%

mutate(surgcasedensity=0) %>%

mutate(instcasedensity=0)

cases<-cases %>%

group_by(surgid) %>%

mutate(surgfirstdate=min(Date)) %>%

ungroup()

cases<-cases %>%

group_by(Institution) %>%

mutate(instfirstdate=min(Date)) %>%

ungroup()

system.time(

for(x in 1:(length(rangebymonth)-1)){

newdatestart=rangebymonth[x]

newdateend=rangebymonth[x+1]

surgtotexpt<-cases %>%

filter(between(Date,firstdate,newdateend)) %>%

count(surgid, name="surgtotexp")

surgcaseexpt<-cases %>%

filter(between(Date,firstdate,newdateend)) %>%

count(surgid, CPT1,name="surgcaseexp")

instcaseexpt<-cases %>%

filter(between(Date,firstdate,newdateend)) %>%

group_by(Institution,CPT1) %>%

count(CPT1, Institution,name="instcaseexp") %>%

ungroup()

#hist case length

histcaselength<-cases %>%

filter(between(Date,firstdate,newdateend)) %>%

group_by(CPT1) %>%

summarize(histcaselength=mean(Patient.In.Room.Duration,na.rm=TRUE))

#hist case length by inst

histcaseinstt<- cases %>%

filter(between(Date,firstdate,newdateend)) %>%

group_by(Institution,CPT1) %>%

summarize(histcaseinst=mean(Patient.In.Room.Duration,na.rm=TRUE))

#hist case length by surgeon

histcasesurgt<- cases %>%

filter(between(Date,firstdate,newdateend)) %>%

group_by(surgid, CPT1) %>%

summarize(histcasesurg=mean(Patient.In.Room.Duration,na.rm=TRUE))

newcases<-cases %>%

filter(Date>=newdatestart) %>%

select(-c(surgtotexp,surgcaseexp,instcaseexp,histcaselength,histcaseinst,histcasesurg,surgtotdensity,surgcasedensity,instcasedensity)) %>%

left_join(surgtotexpt,by="surgid") %>%

left_join(surgcaseexpt,by=c("surgid","CPT1")) %>%

left_join(histcasesurgt,by=c("surgid","CPT1"))%>%

left_join(instcaseexpt,by=c("Institution","CPT1"))%>%

left_join(histcaselength,by="CPT1") %>%

left_join(histcaseinstt,by=c("Institution","CPT1")) %>%

mutate(surgtotdensity=surgtotexp/as.numeric(newdateend-surgfirstdate)) %>%

mutate(surgcasedensity=surgcaseexp/as.numeric(newdateend-surgfirstdate)) %>%

mutate(instcasedensity=instcaseexp/as.numeric(newdateend-instfirstdate)) %>%

bind_rows(cases[(cases$Date>=firstdate & cases$Date<newdatestart),])

cases<-newcases

}

)

#eliminate NA for engineered features

cases$surgtotexp[which(is.na(cases$surgtotexp))]<-0

cases$surgcaseexp[which(is.na(cases$surgcaseexp))]<-0

cases$instcaseexp[which(is.na(cases$instcaseexp))]<-0

cases$histcaselength[which(is.na(cases$histcaselength))]<-0

cases$histcaseinst[which(is.na(cases$histcaseinst))]<-0

cases$histcasesurg[which(is.na(cases$histcasesurg))]<-0

#################################################################

####Above code is for monthly engineered experience features

#################################################################

#################

#Natural Language Processing

#################

tokenizer <- function(x) {

NGramTokenizer(x, Weka_control(min = 1, max = 2))

}

review_corpus = VCorpus(VectorSource(cases$Scheduled.Procedure))

review_corpus = tm_map(review_corpus, content_transformer(tolower))

review_corpus = tm_map(review_corpus, removeWords, c("the", "and", "w", "with", "(actual)", "phi","wrvu","right","left","nbb","endo",stopwords("english")))

replacePunctuation <- content_transformer(function(x) {return (gsub("[[:punct:]]"," ", x))})

removeDash <- content_transformer(function(x) {return (gsub("-","", x))})

review_corpus = tm_map(review_corpus, removeDash)

review_corpus = tm_map(review_corpus, replacePunctuation)

review_corpus = tm_map(review_corpus, stripWhitespace)

expandIOL <- content_transformer(function(x) {return (gsub("iol","intraocular lens", x))})

expandCSection <- content_transformer(function(x) {return (gsub("csection","cesarean section", x))})

expandEGD <- content_transformer(function(x) {return (gsub("egd","esophagogastroduodenoscopy", x))})

expandcath <- content_transformer(function(x) {return (gsub("\\bcath\\b","catheter", x))})

expandcysto <- content_transformer(function(x) {return (gsub("\\bcysto\\b","cystoscopy", x))})

review_corpus = tm_map(review_corpus, expandIOL)

review_corpus = tm_map(review_corpus, expandCSection)

review_corpus = tm_map(review_corpus, expandEGD)

review_corpus = tm_map(review_corpus, expandcath)

review_corpus = tm_map(review_corpus, expandcysto)

inspect(review_corpus[1])

tokenize=tokenizer

review_dtm_tfidf <- DocumentTermMatrix(review_corpus, control = list(weighting=weightTfIdf, tokenize=tokenizer))

review_dtm_tfidf = removeSparseTerms(review_dtm_tfidf, 0.995)

z<-as.matrix(review_dtm_tfidf)

#class conversions

cases$BMI<-as.numeric(as.character(cases$BMI))

cases$Age..Years.<-as.numeric(cases$Age..Years.)

cases$Height<-as.numeric(as.character(cases$Height))

cases$Weight<-as.numeric(as.character(cases$Weight))

#cases$Preop.Creatinine<-as.numeric(as.character(cases$Preop.Creatinine))

#cases$Preop.Hemoglobin<-as.numeric(as.character(cases$Preop.Hemoglobin))

#cases$Preop.Albumin<-as.numeric(as.character(cases$Preop.Albumin))

#cases$Preop.INR<-as.numeric(as.character(cases$Preop.INR))

cases$Institution<-as.factor(cases$Institution)

#convert NULL CPT to 00000

cases$CPT1[which(cases$CPT1=="NULL")]<-"00000"

cases$ASA.Class[which(cases$ASA.Class=="NULL")]<-"-999"

cases$timeofday<-lubridate::hour(as.POSIXct(cases$Anesthesia.Start))

#create data frames to include all relevant variables and NLP matrix

## to run below, you must run the monthly engineered experience features in the above section

atOR<-data.frame(Patient.In.Room.Duration=cases$Patient.In.Room.Duration,surgid=cases$surgid,anesCPT=cases$CPT1,inst=cases$Institution,admittype=cases$Admission.Type,gender=cases$Gender,race=cases$Race,age=cases$Age..Years.,height=cases$Height,weight=cases$Weight,BMI=cases$BMI,ASA=cases$ASA.Class,basemap=cases$Baseline.MAP,emergency=cases$Emergency.Status,holiday=cases$Holiday,weekend=cases$Weekend,service=cases$Surgical.Service,roomtype=cases$Procedure.Room.Type,cardiac=cases$Cardiac.Procedure,anesgen=cases$Anes.Tech.General,creatinine=cases$Preop.Creatinine,hgb=cases$Preop.Hemoglobin,albumin=cases$Preop.Albumin,inr=cases$Preop.INR,CHF=cases$Elixhauser..Congestive.Heart.Failure,arrhythmia=cases$Elixhauser..Cardiac.Arrhythmia,valve=cases$Elixhauser..Valvular.Disease,pvd=cases$Elixhauser..Peripheral.Vascular.Disorders,htncomp=cases$Elixhauser..Hypertension.with.Complications,htnnocomp=cases$Elixhauser..Hypertension.without.Complications,dmcomp=cases$Elixhauser..Diabetes.with.Complications,dmnocomp=cases$Elixhauser..Diabetes.without.Complications,pulm=cases$Elixhauser..Chronic.Pulmonary.Disease,renal=cases$Elixhauser..Renal.Failure,timeofday=cases$timeofday,surgtotexp=cases$surgtotexp,surgcaseexp=cases$surgcaseexp,instcaseexp=cases$instcaseexp,histcaselength=cases$histcaselength,histcaseinst=cases$histcaseinst,histcasesurg=cases$histcasesurg,surgtotdensity=cases$surgtotdensity,surgcasedensity=cases$surgcasedensity,instcasedensity=cases$instcasedensity,z)

#visualize missing data

md.pattern(atOR) #missing data pattern from mice library

atsched<-data.frame(Patient.In.Room.Duration=cases$Patient.In.Room.Duration,surgid=cases$surgid,anesCPT=cases$CPT1,inst=cases$Institution,admittype=cases$Admission.Type,gender=cases$Gender,race=cases$Race,age=cases$Age..Years.,height=cases$Height,weight=cases$Weight,BMI=cases$BMI,ASA=cases$ASA.Class,holiday=cases$Holiday,weekend=cases$Weekend,service=cases$Surgical.Service,roomtype=cases$Procedure.Room.Type,cardiac=cases$Cardiac.Procedure,CHF=cases$Elixhauser..Congestive.Heart.Failure,arrhythmia=cases$Elixhauser..Cardiac.Arrhythmia,valve=cases$Elixhauser..Valvular.Disease,pvd=cases$Elixhauser..Peripheral.Vascular.Disorders,htncomp=cases$Elixhauser..Hypertension.with.Complications,htnnocomp=cases$Elixhauser..Hypertension.without.Complications,dmcomp=cases$Elixhauser..Diabetes.with.Complications,dmnocomp=cases$Elixhauser..Diabetes.without.Complications,pulm=cases$Elixhauser..Chronic.Pulmonary.Disease,renal=cases$Elixhauser..Renal.Failure,timeofday=cases$timeofday,surgtotexp=cases$surgtotexp,surgcaseexp=cases$surgcaseexp,instcaseexp=cases$instcaseexp,histcaselength=cases$histcaselength,histcaseinst=cases$histcaseinst,histcasesurg=cases$histcasesurg,surgtotdensity=cases$surgtotdensity,surgcasedensity=cases$surgcasedensity,instcasedensity=cases$instcasedensity,z)

atincis<-data.frame(Patient.In.Room.Duration=cases$Patient.In.Room.Duration,surgid=cases$surgid,anesCPT=cases$CPT1,inst=cases$Institution,admittype=cases$Admission.Type,gender=cases$Gender,race=cases$Race,age=cases$Age..Years.,height=cases$Height,weight=cases$Weight,BMI=cases$BMI,ASA=cases$ASA.Class,basemap=cases$Baseline.MAP,emergency=cases$Emergency.Status,holiday=cases$Holiday,weekend=cases$Weekend,service=cases$Surgical.Service,roomtype=cases$Procedure.Room.Type,cardiac=cases$Cardiac.Procedure,creatinine=cases$Preop.Creatinine,hgb=cases$Preop.Hemoglobin,albumin=cases$Preop.Albumin,inr=cases$Preop.INR,CHF=cases$Elixhauser..Congestive.Heart.Failure,arrhythmia=cases$Elixhauser..Cardiac.Arrhythmia,valve=cases$Elixhauser..Valvular.Disease,pvd=cases$Elixhauser..Peripheral.Vascular.Disorders,htncomp=cases$Elixhauser..Hypertension.with.Complications,htnnocomp=cases$Elixhauser..Hypertension.without.Complications,dmcomp=cases$Elixhauser..Diabetes.with.Complications,dmnocomp=cases$Elixhauser..Diabetes.without.Complications,pulm=cases$Elixhauser..Chronic.Pulmonary.Disease,renal=cases$Elixhauser..Renal.Failure,timeofday=cases$timeofday,artline=cases$Aterial.Line,anestime=cases$Anesthesia.Duration,block=cases$Anes.Tech.Block,epid=cases$Anes.Tech.Epidural,genanes=cases$Anes.Tech.General,lma=cases$Anes.Tech.LMA,neurax=cases$Anes.Tech.Neuraxial,spinal=cases$Anes.Tech.Spinal,surgtotexp=cases$surgtotexp,surgcaseexp=cases$surgcaseexp,instcaseexp=cases$instcaseexp,histcaselength=cases$histcaselength,histcaseinst=cases$histcaseinst,histcasesurg=cases$histcasesurg,surgtotdensity=cases$surgtotdensity,surgcasedensity=cases$surgcasedensity,instcasedensity=cases$instcasedensity,z)

#################

#Save the processed data

#################

setwd("C:/...") #set your working directory to the location you want to save your files

write.csv(atsched, 'atsched.csv', row.names = FALSE)

write.csv(atOR, 'atOR.csv', row.names = FALSE)

write.csv(atincis, 'atincis.csv', row.names = FALSE)

## **C2. R Code: Training and Testing ML Models**

library(dplyr)

library(lattice)

library(ggplot2)

library(caret)

library(tidyr)

library(h2o) #scalable open source machine learning platform

#load your dataset

setwd(YOUR_DIRECTORY)

your_data<-read.csv("atOR.csv")

your_data<-your_data[ ,2:ncol(your_data)] #removing the additional index from the CSV

#confirm elimination of NA and remove cases greater than 1440 minutes

your_data<-your_data %>%

filter(!is.na(Patient.In.Room.Duration)) %>%

filter(Patient.In.Room.Duration>0) %>%

filter(Patient.In.Room.Duration<1440)

#convert time of day to categorical

your_data$timeofday<-as.factor(your_data$timeofday)

#initialize H2O server

h2o.init(nthreads = -1)

#convert data to H2O format

your_data.h2o<-as.h2o(your_data)

##################

######Train Models

##################

#break data into testing and training sets

trainIndex = sample(1:nrow(your_data), size = round(0.7*nrow(your_data)),replace=FALSE)

train = your_data[trainIndex ,]

test = your_data[-trainIndex ,]

train.h2o<-as.h2o(train)

test.h2o<-as.h2o(test)

y.dep<-"Patient.In.Room.Duration"

x.indep<-setdiff(names(train.h2o), y.dep)

#using your data, build a gbm model to predict in room duration

proclength_gbm <- h2o.gbm(y= y.dep, x = x.indep, training_frame = train.h2o,ntrees=500,nfolds=5,max_depth=5,learn_rate=0.1,stopping_tolerance=0.01,stopping_metric="MAE")

h2o.performance(proclength_gbm)

###saving proclength model

h2o.saveModel(proclength_gbm, path =YOUR_PATH, force = TRUE) #force is to overwrite any existing model; note the new folder I created

#shap summary plot for the model

shap_plot <- h2o.shap_summary_plot(proclength_gbm, train.h2o)

plot(shap_plot)

#local explanation of any predicted output, SHAP explanation shows contribution of features for a given instance.

#shapr works for H2O tree-based models, such as Random Forest, GBM and XGboost only.

shapr_plot <- h2o.shap_explain_row_plot(proclength_gbm, train.h2o, row_index = 1)

plot(shapr_plot)

##################

######Test Above Models

##################

#You can now make predictions of your test data or new data. Using the proclength model as an example:

pred <- h2o.predict(object = proclength_gbm, newdata = test.h2o) #here I use the existing data which was used in training

pred

#you will need to tune your model's hyperparameters using, potentially using the h2o.grid function over hyperparameter ranges

##################

######Train New Models For Quantile Error Model

##################

#using your data and the previous model, build separate gbm models to predict the "Patient.In.Room.Duration" variable

y.dep<-"Patient.In.Room.Duration"

x.indep<-setdiff(names(train.h2o), y.dep)

hyper_params <- list(quantile_alpha = c(.2, .5, .8), ntrees=500,max_depth=5,learn_rate=0.1,stopping_tolerance=0.001,stopping_metric="MAE")

#grid searching can be used for tuning hyperparameters, in this instance we are using it to test quantiles of data

#https://docs.h2o.ai/h2o/latest-stable/h2o-docs/data-science/algo-params/quantile_alpha.html

gridOR <- h2o.grid(x = x.indep, y = y.dep, training_frame = your_data.h2o, algorithm = "gbm",

grid_id = "gridname",

distribution = "quantile",

hyper_params = hyper_params,

nfolds=5)

sortedGrid <- h2o.getGrid("gridname", sort_by = "mae", decreasing = FALSE)

sortedGrid #show models

q5OR<-h2o.getModel(sortedGrid@model_ids[[1]]) #store models separately

q2OR<-h2o.getModel(sortedGrid@model_ids[[2]])

q8OR<-h2o.getModel(sortedGrid@model_ids[[3]])

#prediction interval based on quantile model

h2o.predict(q5OR, your_data)

h2o.predict(q2OR, your_data)

h2o.predict(q8OR, your_data)

#quantile prediction interval output data as a table

quantile_prediction_table->(cbind(h2o.predict(q5OR,new_data),h2o.predict(q2OR,new_data),h2o.predict(q8OR,new_data)))

##################

######Train Models Using automl

##################

#automl is an automated machine learning function found in the H2O package

#auto ML, creating multiple models

regression.auto_inc <- h2o.automl( y = y.dep, x = x.indep, training_frame = train.h2o, max_models=10,nfolds=5,stopping_metric = "MAE",stopping_rounds=15,sort_metric="MAE")

#View the AutoML Leader

regression.auto_inc@leader #this is the leading model using MAE as the sorting metric

#look at all of the models in the leaderboard

lb<-regression.auto_inc@leaderboard

print(lb, n = nrow(lb)) # Print all rows instead of default (6 rows)

#look at all of the models in the leaderboard, adding extra columns: training time, prediction time, algo type

lb2<-h2o.get_leaderboard(object = regression.auto_inc, extra_columns = 'ALL')

lb2

#predict using the leading model

autopred <- h2o.predict(object=regression.auto_inc@leader,newdata=test.h2o)

autopred

#given a trained h2o model, compute the leading model performance on the test dataset

autoperf <- h2o.performance(regression.auto_inc@leader,test.h2o)

autoperf

h2o.mae(autoperf)

h2o.rmse(autoperf)

h2o.r2(autoperf)

#residual analysis

h2o.explain(regression.auto_inc@leader,test.h2o)

#creating a separate model to predict the error in prediction

#using your data and the previous model, build a gbm model to predict the error in prediction from the above gbm model

prederror = h2o.predict(object = proclength_gbm, newdata = your_data.h2o) #predict_proc_length

error_data<-your_data.h2o

error_data$error<-abs(prederror-your_data.h2o["Patient.In.Room.Duration"])

#break data into testing and training sets

trainIndex = sample(1:nrow(your_data), size = round(0.7*nrow(your_data)),replace=FALSE)

train = your_data[trainIndex ,]

test = your_data[-trainIndex ,]

train.h2o<-as.h2o(train)

test.h2o<-as.h2o(test)

y.dep<-"error"

x.indep<-setdiff(names(train.h2o), y.dep)

error_gbm<-h2o.gbm(y=y.dep,x=x.indep,training_frame=train.h2o,stopping_rounds=3,stopping_metric = "MAE",stopping_tolerance=0.01,nfolds = 5,ntrees=500)

###saving error prediction model

h2o.saveModel(error_gbm, path = YOUR_PATH, force = TRUE) #force is to overwrite any existing model; note the new folder created

## **C3. R Code: Making Predictions Using Created Machine Learning Models**

#########

#if you haven’t done this previously, download H2O

#https://docs.h2o.ai/h2o/latest-stable/h2o-docs/downloading.html

if ("package:h2o" %in% search()) { detach("package:h2o", unload=TRUE) }

if ("h2o" %in% rownames(installed.packages())) { remove.packages("h2o") }

pkgs <- c("RCurl","jsonlite")

for (pkg in pkgs) {

if (! (pkg %in% rownames(installed.packages()))) { install.packages(pkg) }

}

install.packages("h2o", type="source", repos=(c("http://h2o-release.s3.amazonaws.com/h2o/latest_stable_R")))

library(h2o)

localH2O = h2o.init()

demo(h2o.kmeans)

#########

library(h2o) #scalable open source machine learning platform

library(dplyr)

h2o.shutdown(prompt = TRUE) #shutting down H2O instance if there is an existing instance running

#load your dataset

setwd("C:/...") #set to location of your files, or use database connection through RODBC

new_data<-read.csv("atOR.csv")

#initialize H2O server

localH2O <- h2o.init(nthreads = -1)

#convert data to H2O format

new_data.h2o<-as.h2o(new_data)

#load preexisting model

proclength_gbm<-h2o.loadModel(MODEL_PATH) #set to location and name of model

error_gbm<-h2o.loadMode(MODEL_PATH) #set to location and name of error prediction model

#prediction of length and error, comparing to actual length and error from prediction, stored in a data frame

pred_proclength<-as.data.frame(h2o.predict(proclength_gbm,new_data.h2o))

names(pred_proclength)[1]<-"pred_proclength"

pred_error<-as.data.frame(h2o.predict(error_gbm,new_data.h2o))

names(pred_error)[1]<-"pred_error"

#local explanation of any predicted output

row<-#your_choice_here

shapr_plot <- h2o.shap_explain_row_plot(proclength_gbm, new_data, row_index = row)

### load existing quantile error prediction models

q5OR<-h2o.loadModel(path = "C:/.../.../GBM_model_R_...") #file name is auto-chosen by H2O

q2OR<-h2o.loadModel(path = "C:/.../.../GBM_model_R_...") #file name is auto-chosen by H2O

q8OR<-h2o.loadModel(path = "C:/.../.../GBM_model_R_...") #file name is auto-chosen by H2O

#prediction interval based on quantile model

a<-h2o.predict(q5OR, your_data)

b<-h2o.predict(q2OR, your_data)

c<-h2o.predict(q8OR, your_data)

#quantile prediction interval output data as a table

quantile_prediction_table->(cbind(a,b,c))

#local explanation of any predicted output

row<-#your_choice_here

shapr_plot <- h2o.shap_explain_row_plot(q5OR, new_data, row_index = row)

# 
